# Whole Genome Sequencing (WGS) Analysis of Virulence and AMR Genes in Extended-Spectrum β-Lactamase (ESBL)-Producing *Escherichia coli* from Animal and Environmental Samples in Four Italian Swine Farms

**DOI:** 10.3390/antibiotics11121774

**Published:** 2022-12-08

**Authors:** Miryam Bonvegna, Laura Tomassone, Henrik Christensen, John Elmerdahl Olsen

**Affiliations:** 1 Department of Veterinary Sciences, University of Turin, Largo P. Braccini 2, 10095 Grugliasco, Italy; 2 Department of Veterinary and Animal Sciences, Faculty of Health and Medical Sciences, University of Copenhagen, Fredriksberg C, 1165 København, Denmark

**Keywords:** antimicrobial resistance, AMR surveillance, next-generation sequencing, One Health

## Abstract

Whole genome sequencing (WGS) is a powerful tool to analyze bacterial genomes rapidly, and can be useful to study and detect AMR genes. We carried out WGS on a group of *Escherichia coli* (*n* = 30), sampled from healthy animals and farm environment in four pigsties in northern Italy. Two × 250bp paired end sequencing strategy on Illumina MiSeq™ was used. We performed in silico characterization of *E. coli* isolates through the web tools provided by the Center for Genomic Epidemiology (cge.cbs.dtu.dk/services/) to study AMR and virulence genes. Bacterial strains were further analyzed to detect phenotypic antimicrobial susceptibility against several antimicrobials. Data obtained from WGS were compared to phenotypic results. All 30 strains were MDR, and they were positive for the genes *bla*_CTX-M_ and *bla*_TEM_ as verified by PCR. We observed a good concordance between phenotypic and genomic results. Different AMR determinants were identified (e.g., *qnrS, sul, tet*). Potential pathogenicity of these strains was also assessed, and virulence genes were detected (e.g., *etsC, gad, hlyF, iroN, iss*), mostly related to extraintestinal *E. coli* pathotypes (UPEC/APEC). However, enterotoxin genes, such as *astA*, *ltcA* and *stb* were also identified, indicating a possible hybrid pathogenic nature. Various replicons associated to plasmids, previously recovered in pathogenic bacteria, were identified (e.g., IncN and IncR plasmid), supporting the hypothesis that our strains were pathogenic. Eventually, through WGS it was possible to confirm the phenotypic antibiotic resistance results and to appreciate the virulence side of our ESBL-producing *E. coli*. These findings highlight the need to monitor commensal *E. coli* sampled from healthy pigs considering a One Health perspective.

## 1. Introduction

*Escherichia coli* is a gram-negative bacterium that normally lives as a commensal in the intestine of humans and animals [1]. Certain strains, however, can be pathogenic, due to the presence of specific virulence factors, and are associated with diseases such as gastroenteritis, urinary tract infections, bloodstream infection and central nervous system diseases [2]. Every year, millions of people suffer from *E. coli*-related maladies worldwide [3,4]. Pathogenic *E. coli* strains cause diseases in both humans and animals. In addition, animals can be carriers of human pathogenic types without presenting clinical signs. In this case, *E. coli* lives as simple commensal microorganism in the intestine of the animals, from where it can possibly spread to humans, other animals and nearby environments.

Pathogenic strains are categorized as either diarrhoeagenic *E. coli* (DEC) or extraintestinal pathogenic *E. coli* (ExPEC). The DEC group encompasses enteropathogenic *E. coli* (EPEC), enterohaemorrhagic *E. coli* (EHEC), enterotoxigenic *E. coli* (ETEC), enteroinvasive *E. coli* (EIEC, including *Shigella*), enteroaggregative *E. coli* (EAEC) and diffusely adherent *E. coli* (DAEC). The ExPEC group is currently categorized into uropathogenic *E. coli* (UPEC), neonatal meningitis *E. coli* (NMEC), sepsis-associated *E. coli* (SEPEC), and avian pathogenic *E. coli* (APEC) [4].

Several studies indicate the presence of multidrug resistant (MDR) and ESBL (extended-spectrum β-lactamase)-pathogenic *E. coli* in livestock [5,6,7]. This is of great concern, since antimicrobial resistance (AMR) is a threat for human and animal health worldwide. A recent study has predicted that by 2050, 10 million humans will die each year due to AMR, if corrective measures are not taken [8]. AMR occurs naturally in microbial populations [9,10]. However, overuse and misuse of antimicrobials in both human and veterinary medicine has greatly contributed to enhancement of the problem [10,11].

Pig production accounts for 32% of consumption of antimicrobials for livestock in Europe. In Italy, 20% of the total amount of antimicrobials are used in swine production [12]. Unfortunately, a frequent use of highest priority, critically important antimicrobials, such as third generation cephalosporins, has been a common practice [12], and this may be linked to the ESBL-producing *E. coli* detection in swine farms [13,14].

Thence, pigs may act as reservoir of ESBL-producing *E. coli*, which can spread to humans via the food chain or through direct contact with animals. Currently, in Italy, data on genetic characterization of ESBL-producing *E. coli* from the swine production sector are scarce, and whole genome sequencing (WGS) is not routinely employed to evaluate virulence and AMR associated genes. In order to understand the genetic background of ESBL-producing *E. coli* strains, and whether such strains have the potential to be pathogenic to humans, in the present research we performed an in-depth WGS analysis of a group of 30 ESBL-producing *E. coli* strains isolated in Italian swine farms from healthy animals and environment.

## 2. Results

### 2.1. ESBL-Producing E. coli Detection

We retrieved a total of 28 positive ESBL-producing *E. coli* from animals and 3 from environmental samples. The majority of positive animal and environmental samples were recovered in Farm G (Table 1). Phenotypic ESBL-producing *E. coli* were confirmed phenotypically through cefpodoxime combination disk test and genotypically through PCR and Sanger sequencing. In all isolates, we detected at least one *bla* gene of the *bla*_CTX-M_ and/or *bla*_TEM_ classes, except for one sample from Farm S. Sanger sequencing revealed that most samples possessed *bla*_CTX-M-1_ (*n* = 19) (Supplementary Table S1). 

### 2.2. Antimicrobial Susceptibility Testing (AST) on Animal Samples

AST analysis revealed that the majority of ESBL-producing *E. coli* strains from animals were MDR, being resistant to more than three antibiotic classes. Resistance against doxycycline, enrofloxacin, florfenicol, tetracycline and trimethoprim/sulfamethoxazole were the most often detected (Table 2). All strains isolated from swine were resistant to doxycycline and tetracycline, with the exception of G1PAE9. The same disseminated resistance was observed against florfenicol. Indeed, 26 out of 27 animal strains were not susceptible to this antibiotic. No strain was resistant to meropenem. However, some strains, such as G1PAE7, were resistant to all tested antibiotics, with the exception of meropenem. 

### 2.3. Presence of AMR Genes

WGS analysis confirmed the presence of both *bla*_CTX-M_ and *bla*_TEM_ genes in all analyzed *E. coli* strains (Table 2). The most commonly detected β-lactamase gene was *bla*_CTX-M-1_ (16/30), followed by *bla*_CTX-M-14_ (12/30), as previously found through Sanger sequencing. The penicillinase *bla*_TEM-1B_ (11/30) was frequently found too. In addition, we detected other 17 acquired resistance genes related to resistance to various antibiotic classes and disinfectants (Table 2, Supplementary Table S2). All *E. coli* had more than one resistance gene, with the majority presenting at least 8 resistance genes. In all strains, we identified *mdf(A)* gene (multiple resistance to benzalkonium chloride, daunomycin, rhodamine). Moreover, 26 of the strains carried *tet* (doxycycline, tetracycline resistance) and *sul* (sulfamethoxazole resistance) gene variants.

### 2.4. Virulence Gene Detection

The genomes were searched for virulence genes which are often expressed by *E. coli* in humans and animals. We detected a wide variety of virulence genes (Table 2, Supplementary Table S3). Some virulence genes were commonly detected, e.g., bacteriocins and microcins encoding genes such as *cea*, and the genetic set composed of *etsC*, *gad*, *hlyF*, *iroN*, *iss*, *iucC*, *iutA, lpfA, mchF, ompT, sitA, terC,* and *traT* (*n* = 14). 

None of the strains were found to carry Shiga-toxin genes. However, enterotoxin genes such as *astA*, *ltcA* and *stb* were observed (Table 2). Other virulence genes detected were *chuA*, coding for an outer membrane hemin receptor (*n* = 4), *gad* expressing a glutamate decarboxylase (*n* = 4), *eilA*, a *Salmonella* HilA homolog (*n* = 3;), *fyuA* expressing a siderophore receptor (*n* = 2;), *hra*, heat-resistant agglutinin gene (*n* = 4), *ireA*, coding for a siderophore receptor (*n* = 1), *irp2*, encoding a non-ribosomal peptide synthetase (*n* = 2), *katP* (*n* = 1), coding for a plasmid-encoded peroxidase, *neuC*, expressing the polysialic acid capsule biosynthetic protein (*n* = 1;), *papC*, (*n* = 1, P1FAE3) encoding outer membrane usher P fimbriae, and *vatA* (*n* = 1) expressing a vacuolating autotransporter toxin. Considering the potential pathogenicity in humans, all strains were predicted to have a probability >80% to be human pathogens based on the arsenal of virulence genes (Supplementary Table S4).

### 2.5. Genetic Relationship between ESBL-Producing Isolates

**Figure 1:**
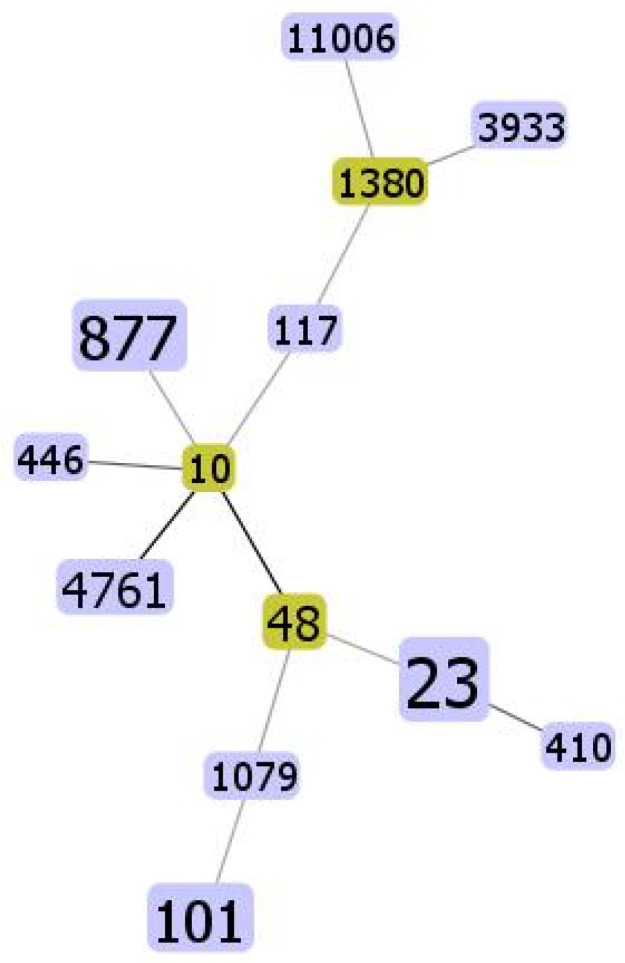
The minimum spanning tree with STs detected across 30 ESBL-producing *E. coli* obtained from pig farms in Italy. An eBURST diagram was created through PHYLOViZ with the goeBURST algorithm. The relation between STs is calculated with only one allelic variation (SLV). STs in yellow squares refer to the “ancestor genotypes”. The size of squares increases with the frequency of recovery.

MLST typing analysis showed that the strains belonged to a wide selection of sequence types (Table 2). The single most common type was ST23 complex (9/30). The other STs common to more strains were ST877 (*n* = 5), ST101 (*n* = 4) and ST48 (*n* = 2). Analysis on the phylogenetic evolution showed that ST23 strains were phylogenetically close to ST48, while ST877 strains were related to the strain with ST10 (Figure 1). 

CH-typing revealed that 8 strains, categorized as ST23, belonged to the CH-category 4-35, while one ST23 strain belonged to the subtype 4-402. CH-type 175-25 was the second most frequently recovered subtype (*n* = 5 strains), while 41-86 was less frequently detected (*n* = 4). The associated O-serotype mostly detected was O8 (*n* = 9), while O45 and O153 were less frequently recovered (*n* = 3) (Table 2).

### 2.6. Prediction of Plasmid Replicons

Plasmid finder predicted more than one plasmid replicon in all strains, with a maximum of seven different plasmid replicons (Table 2). For example, strains P1PAE2, P1PAE4, P1PAE6, P1PAE8 and P1PAE9 showed 7 replicons types: IncB/O/K/Z, IncFlB (AP001918), Incl1-l (Alpha), IncX1, IncY, Col156 and Col (MG828). IncFlB (AP001918) was the most frequently detected plasmid replicon across all strains (*n* = 17). IncB/O/K/Z was less often recovered (*n* = 11). Other detected plasmids were Col 8282, Col 440l, Col (pHAD28), IncFIA (HI1), IncFIB (K), IncFIB (pHCM2), IncFIC (FII), IncFII, IncFII (pCoo), IncQ1, IncR, and IncX3.

## 3. Discussion

In this study, we performed phenotypic susceptibility typing and WGS analysis on ESBL-producing *E. coli* isolated from Italian swine farms. The main focus was on AMR and virulence associated genes, in order to understand if commensal ESBL-producing *E. coli* from healthy pigs could have the potential to be pathogenic to humans and animals and to spread AMR genes with human health impact. Phenotypic tests highlighted the presence of ESBL-producing *E. coli* in the four sampled farms. Although the limited numbers of farms, the prevalence of positive animal samples (28%) was in line with previous European official data, that reported 34% of positive fattening pigs across European countries [15]. However, it was far lower than the Italian official surveillance system, that detected more than 95% of swine samples positive for ESBL-producing *E. coli* in 2019 [15]. The results obtained through phenotypic and genotypic analyses showed that the characterized ESBL-producing *E. coli* were resistant also to antibiotic classes other than beta-lactams. The ESBL and MDR status was confirmed through WGS. 

WGS analysis (see quality parameters’ results in Supplementary Table S5) indicated that the majority of the ESBL-producing strains carried *bla*_CTX-M-1_, responsible for the resistance to extended-spectrum cephalosporins. This agrees with previous studies on European pigs [16,17,18,19]. In a recent survey in the United Kingdom, it was observed that this gene was often associated with *tet* and *sul* genetic variants in the same *E. coli* strain from swine samples [19]. We observed this finding in most of the analyzed strains, too (Supplementary Table S2). WGS also highlighted the presence of resistance genes against other critically important antibiotics such as fluoroquinolones (*qnrS*) and macrolides (*erm*) [20]. Specifically, the *qnrS1* gene, confers low level resistance against fluoroquinolones, making more difficult to treat bacterial pathogens carrying this gene [21,22]. The co-resistance to these antibiotic classes can occur in swine ESBL-positive fecal samples [23,24] and it might be linked to the overuse of fluoroquinolones and macrolides to treat animals in Italy [25,26]. 

Based on the classification of *E. coli* pathotypes, we detected potential UPEC, APEC and hybrid diarrheagenic pathotypes. Considering the acknowledged molecular definition of ExPEC given by Johnson et al. (2003), to classify bacterial strains as ExPEC, isolates need to possess two or more of the following virulence determinants: *papAH*, and/or *papC* (P fimbriae), *sfa-focDE* (S and F1C fimbriae), *afa-draBC* (Dr- binding adhesins), *iutA* (aerobacting siderophore system) and *kpsM II* (group 2 capsules) [27]. Here, we did not detect these genes in the same bacterial strain. However, we recovered some genes, such as *iutA* or *kpsM II*, in association with other virulence markers. *iutA* is involved in the iron metabolism, such as *iroN*, *iucC*, and *sitA*, which were present in the majority of our strains. The bacterial isolates with these virulence markers could be considered commensal, coding for ExPEC-associated virulence genes. Similarly, a reliable classification of UPEC need to consider the presence of at least two or more of the following genes: *chuA*, *fyuA* (coding for ferric yersinia uptake yersiniabactin receptor), *vat* and *yfcV* (adhesin), as stated by Spurbeck et al. (2012) [28]. In our ESBL-producing *E. coli* group, we detected *fyuA* in association with *vat* and *chuA*, only in the finishing-associated *E. coli*, P1FAE3. The other strains did not encode for *chuA*, *vat* and *fyuA* in combination. However, they were positive for one of these genes, in association with other virulence markers (see Table 2). This was observed in G1PAE9 and P1FAE3 strains, in which *fyuA* was present with *irp2*. This last gene is the main virulence determinant in the high pathogenicity island (HPI) [29] of APEC strains, and is considered paramount in avian colibacillosis pathogenicity [30,31]. *irp2* gene encodes a siderophore called iron-repressible protein, involved in yersiniabactin synthesis, which have been recognized in diarrheagenic swine *E. coli* [32]. 

Thence, the majority of our ESBL-producing *E. coli* cannot be confirmed as UPEC pathotypes, even if they encode virulence determinants, frequently recovered in UPEC. Indeed, they often presented the genes *iss*, *ompT* and *traT*, which are typically implicated in extraintestinal pathogenicity. These virulence determinants increase the bacteria survival in serum, blocking complement activity (*iss* and *traT*) and allow survival in urine and resistance to protamine (*ompT*) [33,34,35]. The virulence set of *iroN*, *iss*, *iutA*, *ompT*, and *hlyF* genes, have been considered typical markers of APEC strains too [36]. These five determinants were recovered in 13 of our ESBL-producing *E. coli* strains, mostly recovered in post-weaning animals from two intensive farms. Other markers of extraintestinal pathogenicity, namely *cvaC* and *etsC* [33], were recovered in 13 out of 30 samples, mainly in the post-weaning sector, even at the environmental level (G1PHE2).

*lpfA* was another frequently detected virulence marker. This gene is involved in the expression of an important fimbria for host-cell adhesion. Previous studies detected *lpfA* in EPEC, cattle shiga toxin-producing *E. coli* (STEC), extraintestinal pathogenic *E. coli* and commensal *E. coli* [37,38]. We found a widespread distribution of this gene (19/30) in post-weaning, finishing and sows-associated strains. It was recovered in two environmental samples (G1PHE2 and S1FHE2) as well. All the presumptive ExPEC strains encoded this gene. In addition to ExPEC-suspected strains, other genetic markers, associated to DEC strains, were found. Specifically, the enteroaggregative heat-stable toxin EAST-1, encoded by *astA*, was amplified in animal (G1PAE7, G1PAE8, P1PAE3, TISAE7, T1SAE8) and environmental (S1FHE2) strains. Looking to the strain G1PAE8, the concomitant presence of UPEC-APEC virulence factors (*iroN, iss, iutA, ompT,* and *hlyF*) with *astA*, could indicate a hybrid nature of this particular *E. coli* isolate. In the study of Maluta et al., the same hybrid strains were found in poultry [39]. EAST-1 has been associated with EAEC strains that were isolated in humans and animals [40,41,42]. In swine production, this enterotoxin was detected from diarrheagenic and non- diarrheagenic *E. coli* [42] and its role in pathogenicity in colibacillosis is currently unclear, this might explain EAST-1 detection in our *E. coli* sampled from healthy animals. 

In this case, *stb* was another enterotoxin coding gene present in 2 animal (G1PAE7, P1PAE3) and one environmental (S1FHE2) *E. coli* isolates. The resulting enterotoxin is responsible for secretory diarrhea in human and animal hosts [43]. In our strains, *stb* was always associated to *astA.* In 2 strains carrying *stb*, one animal and the other environmental, *ltcA* gene was also present. This virulence marker codes for a subunit of the heat-labile (LT) enterotoxin and it has been previously detected in ETEC strains from symptomatic post-weaning pigs [44]. All our presumptive DEC presented also *terC* gene, that is implied in tellurium resistance and has been associated to ExPEC and UPEC strains [33].

Other recurrent virulence markers associated to potential DEC were *kpsE* and *kpsMII.* These two genes, involved in bacterial capsule formation, are often sequenced from ExPEC and UPEC strains. For this reason, they are considered genetic markers of ExPEC [33]. Surprisingly, in all our potential DEC strains, these capsule-related determinants were present with enterotoxin-associated genes (*astA*, *stb*). This finding suggests that our strains could be considered hybrid, due to the simultaneous presence of ExPEC and diarrheagenic virulence markers. This is in accordance with the study of Müller et al. (2007), where some *E. coli* pathotypes presented additional virulence genes not traditionally associated to the specific pathogroup [44].

The potential pathogenic nature of our strains is supported by the O-related serotypes detected. Indeed, the serotypes O8 and O45, frequently found in our strains, were previously associated with DEC in piglets and calves [43,44,45]. Moreover, we often detected in one farm the serotype O153, which was found in human ETEC [46]. The majority of our strains (*n* = 9) belonged to the ST23, which, as ST10, is considered a new emerging ExPEC lineage [7].

The finding of plasmid replicons that were previously detected in human clinical samples, also supports the presumptive pathogenicity of our strains. Indeed, one of the most frequently recovered replicons, Col156, was previously found in a plasmid of an *E. coli* clinical strain isolated in Poland (NC009781). Another common plasmid replicon was IncR. This mobile genetic element (MGE)-replicon was recovered from five sows’ samples (as it can be seen in Table 2 for farm G), and it has been previously identified in *Klebsiella pneumoniae* isolated from human urine clinical sample (DQ449578). Another MGE-replicon, IncN, was detected in *E. coli* strains, sampled from four of the five sows from farm T. Plasmids with this replicon were former recovered from *Salmonella enterica* Typhimurium (AY046276), and they are associated to resistance to beta-lactam, streptomycin/spectinomycin and sulphonamides antibiotics [47,48,49]. The plasmid IncX3, previously recovered from an Italian *K. pneumoniae* clinical strain (JN247852), was here detected in a sow strain (T1SAE8). Even the plasmid IncFIB(K), was very similar (98.93%) to the IncFIIk-FIB-like plasmid, previously detected in the Italian *K. pneumoniae* ST258, a pandrug-resistant human clinical isolate [50]. Furthermore, we found some plasmids that were livestock-associated. For example, IncX1, a plasmid already detected in Danish pigs (EU370913), that was sequenced in *E. coli* strains associated to sows (*n* = 4) and post-weaning animals (*n* = 8) (see Supplementary Tables S6 and S7). The conjugative plasmid IncX1 is generally involved in biofilm formation, multidrug efflux and olaquindox (antimicrobial livestock growth promoter) resistance [51]. 

The results from PathogenFinder 1.1 analysis showed that all our strains, sampled from animals or environment, could be considered putative pathogens for human hosts. Indeed, we recovered a high pathogenicity probability (>80%) and relevant virulence markers such as TraF (F plasmid transfer operon, TraF protein), previously sequenced in *S. enterica* Heidelberg (ACF65774), and *Shigella dysenteriae* associated conserved hypothetical protein YhB0 (ABB63325). The highest probabilities of being human pathogens were attributed to isolates from sow samples G1SAE7 (95%) and G1SAE2 (94%), post-weaning and finishing strains (P1PAE4, P1PAE2, P1PAE10, P1FAE1, P1FAE3 = 93%), and an environmental *E. coli* isolate (G1PHE2 = 93%). 

In conclusion, commensal ESBL-producing *E. coli* strains from healthy pigs were found to have the potential to be pathogenic to humans and animals. Some were confirmed pathotypes including UPEC in a strain from finisher and ETEC strains encoding enterotoxin genes *astA*, *ltcA* and *stb*. However, the majority of the isolates did not belong to UPEC or other ExPEC, due to the lack of certain genes that are internationally accepted as determinants [26,27,52]. Thence, it is more correct to say that they are commensal with a set of virulence genes, that are generally recognized in ExPEC pathotypes. However, the fact that we found these genes in isolates from production animals highlights the necessity to monitor these bacteria, as commensal *E. coli* from pigs may serve as reservoir of virulence genes for pathogenic *E. coli*. In this perspective, WGS analysis is a precious tool to understand the real “nature” of confirmed antimicrobial resistant ESBL-producing *E. coli*, even when sampled from healthy animals. WGS enables monitoring the dissemination of specific serotypes/ST-types, that can be transmitted to humans through strict contact with animals or farm environmental contamination. Surveillance of *E. coli* strains present at farm level, will be important to identify new or human-related clones that can disseminate in the swine productive chain. Furthermore, in-silico characterization allows to characterize plasmids that can be common among enteropathogenic bacteria, such as *E. coli* and other important food-borne pathogen (e.g., *Salmonella* spp.). Indeed, as we found for IncN plasmid and other MGEs, the same plasmid can be present in diverse bacterial species. This event can be considered alarming if last-resort antibiotics’ resistance is carried by these plasmids. For all these reasons, we support the use of this new monitoring approach at farm level, especially in intensive production, where high number of animals are present, and spread of certain *E. coli* strains can be facilitated by poor hygiene conditions and lack of biosecurity measures. 

## 4. Materials and Methods

### 4.1. Animal and Environmental Sampling

We performed animal (fecal) and environmental (farm barns) sampling in 4 farms (farm G, P, S and T, named according to owners’ surname) in the Piedmont region (northern Italy) from October 2019 to September 2020. The farms represented the standard swine farms of the area and were selected considering the willingness of the farmer to participate. We collected 30 swab samples from pigs at three production stages (finishing, post-weaning, sows) in each farm, with the exception of farm S, where only 10 samples were taken (finishing was the only production stage in this farm). The sampled pigs were healthy at the time of the survey as determined by clinical inspection. Six environmental samples were collected from barn walls and floor in each farm (only two in farm S). Swabs were transported to the laboratory in Amies transport medium. 

### 4.2. Bacterial Strains and Antimicrobial Susceptibility Testing

The isolation of ESBL-producing *E. coli* was carried out following the protocol by Hasman et al. (2015), however with incubation temperature of 37 °C [53]. Colonies growing on MacConkey agar (Oxoid, Wade Road Basingstoke, UK) with 1 mg/L cefotaxime (Sigma-Aldrich, St. Louis, MO, USA) were purified as single colonies on Blood agar (Oxoid Ltd., Wade Road Basingstoke, UK). After purification, Matrix-assisted laser desorption/ionisation time-of-flight mass spectrometry (MALDI-TOF/MS) (microflex Biotyper^®^ LT. Bruker Daltonics GmbH, Bremen, Germany), was used to confirm *E. coli*, selecting one or two colonies per plate. After recovering ESBL-producing *E. coli* in animals and from the environment [53], we selected 27 strains from pigs at different stages of the production: finishing (*n* = 3), post-weaning phase (*n* = 14) and sows (*n* = 10). Environmental samples (*n* = 3) were taken from finishing (*n* = 1) and post-weaning sectors (*n* = 2). All the strains were confirmed as ESBL through cefpodoxime combination disk test (Oxoid, Wade Road Basingstoke, UK) and by PCR directed against *bla*_CTX-M_ and *bla*_TEM_ (see below). 

Antibiotic susceptibility test (AST) was performed by Kirby-Bauer disc diffusion method only on animal samples to detect phenotypic resistance to doxycycline, enrofloxacin, florfenicol, gentamicin, tetracycline, trimethoprim/sulfamethoxazole and meropenem (Oxoid Ltd., Basingstoke, UK). The evaluation of inhibition zones followed the criteria of the Clinical and Laboratory Standards Institute (CLSI VET 08) [54] for all antibiotics, with the exception of meropenem, for which the susceptibility was tested following the European Committee on Antimicrobial Susceptibility Testing (EUCAST v11.0) [55]. Quality control of method was based on parallel testing of isolates from Turin University Culture Collection (http://www.tucc.unito.it/en, accessed on 10 December 2021).

### 4.3. PCR Methods and Analysis of PCR Products

DNA was extracted from pure colonies with a modified boiling method [56]. The spectrophotometer NanoDrop™ 2000 (Thermo Scientific, Waltham, WA, USA), was used to measure the quantity of extracted DNA according to published protocol [57]. PCR, targeting the β-lactamase genes *bla*_CTX-M_ and *bla*_TEM_, was performed using primer sequences previously used by Hasman et al. (2005) and Jiang et al. (2005) [58,59]. The amplified DNA was purified with ExoSAP-IT™ PCR Product Clean-up Kit (GE Healthcare Limited, Chalfont, UK) and Sanger sequenced at BMR Genomics institute (Padova, Italy) using forward primer as sequence primer. 

BioEdit 7.2.5 Sequence Alignment Editor© software was used to analyze the resulting nucleotide sequences. ClustalW tool was set up for multiple alignment of sequences with reference bacterial genome sequences. We used the *E. coli* strain CFS3313 (CP026941.2) for *bla*_CTX-M-1_ gene, the strain I1-1 (KY095111.1) for *bla*_CTX-M-14_ and the strain EcPF5 (CP054237.1) for *bla*_TEM-1_. BLAST^®^ (https://blast.ncbi.nlm.nih.gov/Blast.cgi, accessed on 5 January 2022) was run to compare our sequences with sequences in the GenBank database.

### 4.4. Whole Genome Sequencing and Analysis of Sequences

Whole genome sequencing (WGS) was performed to determine Multi locus sequence type (MLST), *fumC_fimH* (CH-type), plasmid replicons, antimicrobial resistance genes, virulence genes, and to assess serotype. Briefly, DNA was extracted with GeneJet Genomic DNA purification kit (Thermo Scientific™, Waltham, WA, USA). RNAse treatment was performed during DNA extraction with an endoribonuclease (Thermo Scientific™, USA) and library preparation was carried out with Nextera XT (Illumina, San Diego, CA, USA) according to the recommendation of the supplier. Extracted samples were eluted in sterile DNA-free water. Quality parameters were assessed with NanoDrop, ensuring a ratio of absorbance 260:280 equal or higher than 1.8, and a quantity of around 30 ng/µL. In order to perform WGS analysis, 2 × 250 bp paired end sequencing strategy on Illumina MiSeq™ (Illumina, San Diego, CA, USA) was used. Assembly of raw reads was performed with CLC Genomic Workbench v. 20.0.4 (Qiagen, Aarhus, Denmark). The cut-off for contig size was 1000. Trimming was performed in CLC with 0.01 error probability. The coverage was set to be minimum 30× [60]. 

After assembly of contigs, the *E. coli* genomes were analyzed using Center for Genomic Epidemiology (CGE) tools (cge.cbs.dtu.dk/services/) [61]. ResFinder 4.1 was used to detect acquired resistance genes, with a threshold of identity of 85% and a minimum length of 60%. Virulence genes were identified through VirulenceFinder 2.0. PlasmidFinder 2.1 was run to detect plasmid replicons, while MLST 2.0 was used to type our strains. SerotypeFinder 2.0 was used to predict the serotype, and CHTyper-1.0 was launched to detect the house-keeping genes *fumC*, coding for fumarase enzyme, and *fimH*, coding for a specific adhesin (type 1 fimbriae), to further categorise the *E. coli* strains. The probability of our strains to have human-associated pathogenicity was estimated using PathogenFinder 1.1. *E. coli* pathotype was assessed based on the classification used in Johnson et al. (2003) [27]. Using MLST results, a minimum spanning tree was produced using PHYLOViZ with the goeBURST algorithm [62].

## Figures and Tables

**Table 1:** Number of ESBL-producing *E. coli* isolated in animal (fecal) and environmental samples from swine farms located in Northern Italy. The farm ID (G, P, S and T) recalls the farmer’s surname.

Farm ID	Animal	Environment
	*n* positive samples/*n* tested (%; 95% CI)	
G	12/30 (40%; 22.5, 57.5)	2/6 (33.3%; 0, 71)
P	11/30 (36.7%; 19.4, 53.9)	0/6 (0%; 0, 39.3)
S	0/10 (0%; 0, 25.9)	1/2 (50%; 0, 100)
T	5/30 (16.7%; 8.2, 38.5)	0/6 (0%; 0, 39.3)
Total	28/100 (28%; 19.2, 36.8)	3/20 (15%; 0, 30.6)

**Table 2:** The phenotypic and genetic features of ESBL-producing *E. coli* isolates from pigs of finishing (F), post-weaning (P), sows (S) groups of four farms (G, P, S, and T). A column with the AMR phenotype was added. Abbreviations: DOX, doxycycline. ENR, enrofloxacin. FLO, florfenicol. GEN, gentamycin. SXT, trimethoprim/sulfamethoxazole. TET, tetracycline. n.a., not applicable (environmental strains not tested due to economic reasons). ST4767 *:is the nearest ST found for the strain T1SAE10.

Isolate	MLST Type	O-Serotype	CH-Type	β-Lactamase (*bla*) Genes	AMR Genes	AMR Phenotype	Plasmid Replicon	Virulence Genes
G1PAE2	ST101	O153	41-86	CTX-M-1 TEM-1C	*aadA1, aadA2b catA1, cmlA1, mdf(A),* *sitABCD, sul3, tet(A)*	DOX, FLO, TET, SXT	IncFlB (AP001918), IncFIA, IncFIC (FII), Incl1-l (Alpha), IncX1	*cma, cvaC* *etsC* *hlyF* *iroN* *iss* *iucC* *iutA* *lpfA* *mchF* *ompT* *sitA* *terC* *traT*
G1PAE3	ST101	O153	41-86	CTX-M-1, TEM-1C	*aadA1, aadA2b catA1, cmlA1, mdf(A),* *sitABCD, sul3, tet(A)*	DOX, FLO, TET, SXT	IncFlB (AP00118), IncFIA, IncFIC (FII), Incl1-l (Alpha), IncX1, Col (MG828)	*cvaC* *etsC* *hlyF* *iroN* *iss* *iucC* *iutA* *lpfA* *mchF* *ompT* *sitA* *terC, traT*
G1PAE4	ST101	O153	41-86	CTX-M-1, TEM-1C	*aadA1, catA1, cmlA1, aadA2b, mdf(A),* *sitABCD, sul3, tet(A)*	DOX, FLO, TET, SXT	IncFlB (AP001918), IncFIA, IncFIC (FII), Incl1-l (Alpha), IncX1, Col (MG828)	*cma cvaC* *etsC* *hlyF* *iroN* *iss* *iucC* *iutA* *lpfA* *mchF* *ompT* *sitA* *terC* *traT*
G1PAE7	ST11006	O17	3-143	CTX-M-14, TEM-1B	*aac(6′)-lb-cr, aac(6′)-lb3, cmlA1, erm(B), mdf(A), mph(A), tet(B)*	DOX, ENR, FLO, GEN, TET, SXT	IncB/O/K/Z, IncFlB (AP001918), IncFII (pHN7A8)	*asta* *chuA* *eilA* *kpsE* *kpsMII* *stb* *terC* *traT*
G1PAE8	ST1079	O6	19-32	CTX-M-1, TEM-1B	*aacA1, aadA2b, aph(3′′)-lb, aph(6)-lb, catA1, cmlA1, mdf(A),* *sitABCD,* *sul3, sul2, tet(A)*	DOX, ENR, FLO, TET, SXT	IncFlB (AP001918), IncFIC(FII), Incl1-l (Alpha), IncY	*asta* *cea, cvaC* *etsC* *hlyF, hra* *iroN* *iss* *iucC* *iutA* *lpfA* *mchF* *ompT* *sitA* *terC* *traT*
G1PAE9	ST23	O78	4-402	CTX-M-1	*aadA1, catA1, cmlA1, aadA2b, mdf(A),* *sitABCD, sul3*	ENR, FLO, SXT	IncFlB (AP001918), IncFIC(FII), Incl1-l (Alpha)	*fyuA, hlyF, irp2* *iss* *iucC* *iutA* *lpfA* *ompT* *sitA* *terC*
G1PHE1	ST10	unknown	11-54	CTX-M-15	*mdf(A), qnrS1, tet(B)*	na	Col (MG828), IncFIB (pHCM2), Incl1-l (Alpha)	*cea* *gad* *terC*
G1PHE2	ST23	O8	4-35	CTX-M-1	*aadA1, dfrA12 catA1, cmlA1, mdf(A),* *sitABCD, sul3*	na	Col440ll, IncFlB (AP001918), IncFIC(FII), Incl1-l (Alpha), IncY	*cea* *cia* *cvaC* *etsC* *hlyF* *iroN* *iss* *iucC* *iutA* *lpfA* *mchF* *ompT* *sitA* *terC* *traT*
G1SAE2	ST877	unknown	175-25	CTX-M-1	*aadA1, catA1, cmlA1, dfrA12, mdf(A), sul3, sul1, tet(B)*	DOX, ENR, FLO, TET, SXT	IncR, IncFII, Col (pHAD28)	*cea, hra* *ompT* *terC, traT, tsh*
G1SAE4	ST877	O45	175-25	CTX-M-1	*aph(3′′)-lb, aph(6′)-ld, cmlA1, aadA1, aadA2b, mdf(A),* *sul3, tet(A)*	DOX, ENR, FLO, TET, SXT	IncR, IncFII, Col (pHAD28), IncFIB (pHCM2)	*lpfA* *ompT* *terC, traT, tsh*
G1SAE8	ST877	O45	175-25	CTX-M-1	*aph(3′’)-lb, aph(6′)-ld, cmlA1, aadA1, aadA2b, mdf(A),* *sul3, tet(A)*	DOX, ENR, FLO, TET	IncR, IncFII, Col (pHAD28), IncFIB (pHCM2)	*lpfA* *ompT* *terC, traT, tsh*
G1SAE7	ST877	O45	175-25	CTX-M-1	*aph(3′’)-lb, aph(6′)-ld, cmlA1, aadA1, aadA2b, mdf(A),* *sul3, tet(A)*	DOX, ENR, FLO, TET, SXT	IncR, Col (pHAD28), IncFIB (pHCM2)	*lpfA* *ompT* *terC*
G1SAE10	ST877	O45	175-25	CTX-M-1	*aph(3′’)-lb, aph(6′)-ld, cmlA1, aadA1, aadA2b, mdf(A),* *sul3, tet(A)*	DOX, ENR, FLO, TET, SXT	IncR, IncFII, Col (pHAD28), IncFIB (pHCM2)	*lpfA* *ompT* *terC, traT, tsh*
P1FAE1	ST101	O88	41-86	CTX-M-14, TEM-1B	*aac(6′)-Ib3, aac(6′)-Ib-cr, aadA1, cmlA1,* *ermB, dfrA1, mdf(A), mph(A), sitABCD, sul3, tet(A)*	DOX, ENR, FLO, TET, SXT	IncB/O/K/Z, IncFlB (AP001918), IncFIC(FII), Incl1-l (Alpha)	*cvaC* *etsC* *hlyF* *iroN* *iss* *iucC* *iutA* *lpfA* *mchF* *ompT* *sitA* *terC, traT* *tsh*
P1FAE3	ST117	O9	45-97	CTX-M-14	*aac(6′)-ib3, aac(6′)-Ib-cr aph(3′’)-lb, aph(6)-ld, cmlA1, ermB, dfrA1, mdf(A), mph(A),* *sitABCD, sul2, tet(B)*	DOX, FLO, TET, SXT	IncB/O/K/Z, IncFlB (AP001918)	*chuA, cia, cvaC* *etsC* *fyuA* *hlyF, ireA* *iroN, irp2* *iss* *iucC* *iutA, katP,* *lpfA* *mchF* *ompT* *papC,* *sitA* *terC, traT, vat*
P1FAE7	ST446	unknown	7-41	CTX-M-1, TEM-1A	*aac(3)-IV, aadA1, aadA2b, aadA5, aph(3′)-la, aph(4)-la, catA1, cmlA1, dfrA12, dfrA17, mdf(A), mph(B),* *sul3, tet(B)*	DOX, ENR, FLO, TET,	IncB/O/K/Z, IncFlB (AP001918), Incl1-l(Alpha)	*terC*
P1PAE2	ST23	O8	4-35	CTX-M-14, TEM-1B	*aac(6′)-ib-cr, aac(6′)-Ib3, aadA1, aadA5, cmlA1, dfrA17, ermB, mdf(A), mph(A),* *qnrS1, sitABCD, sul2, tet(A)*	DOX, ENR, FLO, TET, SXT	IncB/O/K/Z, IncFlB (AP001918), Incl1-l (Alpha), IncX1, IncY, Col156, Col (MG828)	*cea, celb* *cia cib* *cvaC* *etsC* *hlyF* *iroN* *iss* *iucC* *iutA* *lpfA* *mchF* *ompT* *sitA* *terC* *traT*
P1PAE3	ST3933	O7	506-544	CTX-M-14, TEM-1B	*aac(6′)-Ib-cr, aac(6′)-Ib3, cmlA1, ermB, mdf(A), mph(A),* *qnrS1, sitABCD, tet(M)*	DOX, ENR, FLO, TET, SXT	IncB/O/K/Z, IncFlB (AP001918), IncFIA, IncFIC(FII), IncX1	*astA* *chuA* *eilA* *kpsE* *kpsMII_K5* *ltcA* *sitA* *stB* *terC* *traT*
P1PAE4	ST23	O8	4-35	CTX-M-14, TEM-1B	*aac(6′)-Ib-cr, aac(6′)-Ib3, aadA1, aadA5, cmlA1, ermB1, dfrA17, mdf(A), mph(A),* *qnrS1, sitABCD, sul2, tet(A)*	DOX, ENR, FLO, TET, SXT	IncB/O/K/Z, IncFlB (AP001918), Incl1-l (Alpha), IncX1, IncY, Col156, Col (MG828)	*cea* *celb* *cia* *cib* *cvaC* *etsC* *gad* *hlyF* *iroN* *iss* *iucC* *iutA* *lpfA* *mchF* *ompT* *sitA* *terC* *traT*
P1PAE6	ST23	O8	4-35	CTX-M-14, TEM-1B	*aac(6′)-Ib-cr, Aac(6′)-Ib3, aadA1, aadA5, cmlA1, ermB, dfrA17, mdf(A), mph(A),* *qnrS1, sitABCD, sul2*	DOX, ENR, FLO, TET, SXT	IncB/O/K/Z, IncFlB (AP001918), Incl1-l (Alpha), IncX1, IncY, Col156, Col (MG828)	*cea* *celb* *cia* *cib* *cvaC* *etsC* *hlyF* *iroN* *iss* *iucC* *iutA* *lpfA* *mchF* *ompT* *sitA* *terC* *traT*
P1PAE7	ST23	O8	4-35	CTX-M-14, TEM-1B	*aac(6′)-Ib3, Aac(6′)-Ib-cr aadA1, aadA5, cmlA1, ermB, dfrA17, mdf(A), mph(A),qnrS1,* *sitABCD, sul2, tet(A)*	DOX, ENR, FLO, TET, SXT	IncB/O/K/Z, IncFlB (AP001918), Incl1-l (Alpha), IncX1, IncY, Col156	*cea* *celb* *cia* *cvaC* *etsC, gad* *hlyF* *iroN* *iss* *iucC* *iutA* *lpfA* *mchF* *ompT* *sitA* *terC* *traT*
P1PAE8	ST23	O8	4-35	CTX-M-14, TEM-1B	*aac(6′)-Ib3, aac(6′)-Ib-cr aadA1, aadA5, cmlA1, ermB, dfrA17, mdf(A), mph(A),* *qnrS1, sitABCD, sul2, tet(A)*	DOX, ENR, FLO, TET, SXT	IncB/O/K/Z, IncFlB (AP001918), Incl1-l (Alpha), IncX1, IncY, Col156, Col (MG828)	*cea* *celb* *cia* *cib* *cvaC* *etsC* *hlyF* *iroN* *iss* *iucC* *iutA* *lpfA* *mchF* *ompT* *ompT* *sitA* *terC* *traT*
P1PAE9	ST23	O8	4-35	CTX-M-14, TEM-1B	*aac(6′)-Ib3, aac(6′)-Ib-cr, aadA1, aadA5, cmlA1, ermB, dfrA17, mdf(A), mph(A),* *qnrS1, sitABCD, sul2, tet(A)*	DOX, ENR, FLO, TET, SXT	IncB/O/K/Z, IncFlB (AP001918), Incl1-l (Alpha), IncX, Col156, Col (MG828)	*cea* *celb* *cia* *cib* *cvaC* *etsC* *hlyF* *iroN* *iss* *iucC* *iutA* *lpfA* *mchF* *ompT* *sitA* *terC* *traT*
P1PAE10	ST23	O8	4-35	CTX-M-14, TEM-1B	*aac(6′)-Ib3, aac(6′)-Ib-cr aadA1, aadA5, cmlA1, ermB, dfrA17, mdf(A), mph(A),* *qnrS1, sitABCD, sul2, tet(A)*	DOX, ENR, FLO, TET, SXT	IncB/O/K/Z, IncFlB (AP001918), Incl1-l (Alpha), IncX1, Col156, Col (MG828)	*cea* *celb* *cia* *cvaC* *etsC* *hlyF* *iroN* *iss* *iucC* *iutA* *lpfA* *mchF* *ompT* *sitA* *terC, traT*
S1FHE2	ST1380	O17	35-47	CTX-M-14	*aadA2 cmlA1, dfrA12, dfrA36, floR, mdf(A), qnrS1, sul1, sul2*	na	Col8282, IncQ1, IncFlB (AP001918), IncFlC (Fll), IncFll (pCoo), IncY	*astA* *chuA* *eilA* *hra* *kpsE* *kpsMII* *lpfA* *ltcA* *stb* *terC* *traT*
T1SAE6	ST4761	O107	252-27	CTX-M-1	*aadA1, aadA2 cmlA1, dfrA12, mdf(A), mph(A), sul3, tet(A)*	DOX, FLO, TET, SXT	IncN, IncX1	*kpsE* *kpsMII* *terC*
T1SAE7	ST48	O61	11-0	CTX-M-1, TEM-1B	*aadA1, aadA2 cmlA1, dfrA12, mdf(A), mph(A), sul3, tet(A)*	DOX, ENR, FLO, TET, SXT	IncN, Col (MG28, IncFIA (Hl1), Inc FIB (K), IncX1	*astA* *gadA* *hra* *iss* *neuC* *papC* *terC*
T1SAE8	ST48	O8	11-54	SHV-12, TEM-1A	*mdf(A), mph(B), qnrS1, tet(B)*	DOX, ENR, TET,	IncX3, IncY, Col440l	*astA* *gad* *terC*
T1SAE9	ST410	O25	4-24	CTX-M-1	*aadA1, aadA2 cmlA1, dfrA12, mdf(A), mph(A), sul3, tet(A)*	DOX, ENR, FLO, TET, SXT	IncN, IncX1	*lpfA* *terC*
T1SAE10	ST4767 *	O107	252-27	CTX-M-1	*aadA1, aadA2 cmlA1, dfrA12, mdf(A), mph(A), sul3, tet(A)*	DOX, ENR, FLO, TET, SXT	IncN, IncX1	*gad* *kpsE* *kpsMII* *terC*

## Data Availability

Not applicable.

## Supplementary Materials

The following supporting information can be downloaded at: https://www.mdpi.com/article/10.3390/antibiotics11121774/s1, Supplementary Table S1: β-lactamase genes detected by PCR, Sanger sequencing and WGS from ESBL-producing *E. coli* (*n* = 31) sampled from healthy pigs and farm environment, northern Italy. Supplementary Table S2: AMR associated genes, with relative function and frequency, detected by WGS in ESBL-producing *E. coli* sampled from healthy pigs and farm environment, northern Italy. Supplementary Table S3: virulence genes, with relative function and frequency, detected by WGS in ESBL-producing *E. coli* sampled from healthy pigs and farm environment, northern Italy. Supplementary Table S4: potential human pathogenicity, expressed as percentage, of ESBL-producing *E. coli* sampled from healthy pigs and farm environment, northern Italy. The results were obtained through PathogenFinder 1.1 (https://cge.cbs.dtu.dk/services/, accessed on 11 December 2021).; Supplementary Table S5: WGS quality parameters of all analysed *E. coli* strains. Supplementary Table S6: identification of virulence and AMR genes on plasmids located on specific contigs of G1PAE2 genome. To confirm the presence of virulence and/or AMR genes on particular plasmids, tools (PlasmidFinder, ResFinder and VirulenceFinder) from Center for Genomic Epidemiology (https://cge.cbs.dtu.dk/services/, accessed on 1 December 2021) were used. Supplementary Table S7: comparison of plasmid-positive contigs found in G1PAE2 *E. coli* strain with sequences recorded in BLAST ^®^ database (https://blast.ncbi.nlm.nih.gov/Blast.cgi, accessed on 18 November 2022).
