# Supplementary material for: Whole Genome Sequencing (WGS) Analysis of Virulence and AMR Genes in Extended-Spectrum β-Lactamase (ESBL)-Producing Escherichia coli from Animal and Environmental Samples in Four Italian Swine Farms
Source: Antibiotics (Basel). 2022 Dec 8;11(12):1774. doi: 10.3390/antibiotics11121774 (PMC9774568; doi:10.3390/antibiotics11121774)
Supplement: Supplementary file 1 [file antibiotics-11-01774-s001.zip › antibiotics-1914485-supplementary.pdf]

Supplementary files

Supplementary Table S1: β-lactamase genes belonging to *bla*$_{CTX-M}$ and *bla*$_{TEM}$ type detected by PCR, Sanger sequencing and WGS from ESBL *E. coli* (n=31) sampled from healthy pigs and farm environment, northern Italy. The sample G1PAE6 was only tested through PCR and Sanger sequencing. *S1FHE2 resulted positive for *bla*$_{CTX-M-14}$ by WGS and not by PCR.

Sample	Source	*bla*$_{CTX-M}$	*bla*$_{TEM}$
G1PAE2	weaned pig	*bla*$_{CTX-M-1}$	*bla*$_{TEM-1}$
G1PAE3	weaned pig	*bla*$_{CTX-M-1}$	*bla*$_{TEM-1}$
G1PAE4	weaned pig	*bla*$_{CTX-M-1}$	*bla*$_{TEM-1}$
G1PAE6	weaned pig	*bla*$_{CTX-M-1}$	neg
G1PAE7	weaned pig	*bla*$_{CTX-M-1}$	*bla*$_{TEM-1}$
G1PAE8	weaned pig	*bla*$_{CTX-M-1}$	*bla*$_{TEM-1}$
G1PAE9	weaned pig	*bla*$_{CTX-M-1}$	neg
G1PHE1	farm environment	*bla*$_{CTX-M-15}$	neg
G1PHE2	farm environment	*bla*$_{CTX-M-1}$	neg
G1SAE2	sow	*bla*$_{CTX-M-1}$	neg
G1SAE4	sow	*bla*$_{CTX-M-1}$	neg
G1SAE7	sow	*bla*$_{CTX-M-1}$	neg
G1SAE8	sow	*bla*$_{CTX-M-1}$	neg
G1SAE10	sow	*bla*$_{CTX-M-1}$	neg
P1PAE2	weaned	*bla*$_{CTX-M-14}$	*bla*$_{TEM-1}$

P1PAE3	weaned	*bla*$_{CTX-M-14}$	*bla*$_{TEM-1}$
P1PAE4	weaned	*bla*$_{CTX-M-14}$	*bla*$_{TEM-1}$
P1PAE6	weaned pig	*bla*$_{CTX-M-14}$	*bla*$_{TEM-1}$
P1PAE7	weaned pig	*bla*$_{CTX-M-14}$	*bla*$_{TEM-1}$
P1PAE8	weaned pig	*bla*$_{CTX-M-14}$	*bla*$_{TEM-1}$
P1PAE9	weaned pig	*bla*$_{CTX-M-14}$	*bla*$_{TEM-1}$
P1PAE10	weaned pig	*bla*$_{CTX-M-14}$	*bla*$_{TEM-1}$
P1FAE7	finishing pig	*bla*$_{CTX-M-1}$	*bla*$_{TEM-1}$
P1FAE3	finishing pig	*bla*$_{CTX-M-14}$	neg
P1FAE1	finishing pig	*bla*$_{CTX-M-14}$	*bla*$_{TEM-1}$
S1FHE2	farm environment	*bla*$_{CTX-M-14*}$	neg
T1SAE6	sow	*bla*$_{CTX-M-1}$	neg
T1SAE7	sow	*bla*$_{CTX-M-1}$	*bla*$_{TEM-1}$
T1SAE8	sow	neg	*bla*$_{TEM-1}$
T1SAE9	sow	*bla*$_{CTX-M-1}$	neg
T1SAE10	sow	*bla*$_{CTX-M-1}$	neg

Supplementary Table S2: AMR associated genes, with relative function and frequency, detected by WGS in ESBL *E. coli* sampled from healthy pigs and farm environment, northern Italy.

**AMR gene**	**Function**	**Number of strains with the gene and frequency (%)**
aac(6')-Ib-cr	Fluoroquinolones and aminoglycosides resistance	10; 33.3%
aac(6')-Ib3	Amikacin, tobramycin resistance	10; 33.3%
aph(3'')-Ib	Aminoglycoside resistance	6; 20%
aph(6)-Id	Aminoglycoside resistance	1; 3.3%
aph(6')-Id	Aminoglycoside resistance	4; 13.3%
aph(4)-Ia	Aminoglycoside resistance	1; 3.3%
aadA1	Aminoglycoside resistance	23; 76.7%
aadA2	Aminoglycoside resistance	5; 16.7%
aadA2b	Aminoglycoside resistance	10; 33.3%
aadA5	Aminoglycoside resistance	8; 26.7%
$bla_{CTX\text{-}M\text{-}1}$	Beta-lactam resistance	16; 53.3%
$bla_{CTX\text{-}M\text{-}14}$	Beta-lactam resistance	11; 36.7%
$bla_{CTX\text{-}M\text{-}15}$	Beta-lactam resistance	1; 3.3%
$bla_{TEM\text{-}1A}$	Beta-lactam resistance	2; 6.7%
$bla_{TEM\text{-}1B}$	Beta-lactam resistance	12; 40%
$bla_{TEM\text{-}1C}$	Beta-lactam resistance	3; 10%

catA1	Chloramphenicol resistance	8; 26.7%
cmlA1	Phenicol resistance	28; 93.3%
dfrA12	Trimethoprim resistance	8; 26.7%
dfrA17	Trimethoprim resistance	8; 26.7%
dfrA36	Trimethoprim resistance	1; 3.3%
erm(B)	Macrolide resistance	1; 3.3%
floR	Phenicol resistance	1; 3.3%
mdf(a)	Multiple resistance to benzalkonium chloride, daunomycin, rhodamine	30; 100%
mph(A)	Macrolide resistance	16; 53.3%
mph(B)	Macrolide resistance	2; 6.7%
qnrS1	Quinolone resistance	11; 36.7%
sitABCD	Peroxide hydrogen resistance	16; 53.3%
sul1	Sulphonamide resistance	2; 6.7%
sul2	Sulphonamide resistance	10; 33.3%
sul3	Sulphonamide resistance	17; 56.7%
tet(A)	Tetracycline resistance	19; 63.3%
tet(B)	Tetracycline resistance	6; 20%

Supplementary Table S3: virulence genes, with relative function and frequency, detected by WGS in ESBL *E. coli* sampled from healthy pigs and farm environment, northern Italy.

**Virulence gene**	**function**	**Number of strains with the gene and frequency (%)**
astA,	EAST-1 heat-stable toxin	6; 20%
cea	colicin E1	11; 36.7%
celb,	Endonuclease colicin E2	7; 23.3%
chua	Outer membrane hemin receptor	4; 13.3%
cia	Colicin ia	10; 33.3%
cib	Colicin ib	5; 16.7%
cma	Colicin M	1; 3.3%
cvaC,	Microcin C	14; 46.7%
eilA	Salmonella HilA homolog	3; 10%
etsC	Putative type I secretion outer membrane protein	14; 46.7%
fyuA	Siderophore receptor	2; 6.7%
gad	Glutamate decarboxylase	6; 20%
hlyF	Hemolysin F	15; 50%
hra	Heat-resistant agglutinin	4; 13.3%
ireA	Siderophore receptor	1; 3.3%
iroN,	Enterobactin siderophore receptor protein	14; 46.7%
Irp2	High molecular weight protein 2 non-ribosomal peptide synthetase	2; 6.7%
iss	Increased serum survival	16; 53.3%
iucC	Aerobactin synthetase	15; 50%

iutA,	Ferric aerobactin receptor	15; 50%
katP	Plasmid-encoded catalase peroxidase	1; 3.3%
kpsE,	Capsule polysaccharide export inner-membrane protein	5; 16.7%
kpsMII,	Polysialic acid transport protein; Group 2 capsule	5; 16.7%
kpsMII_K5	Polysialic acid transport protein; Group 2 capsule	1; 3.3%
lpfA	Long polar fimbriae	21; 70%
ltcA	Heat-labile enterotoxin A subunit	2; 6.7%
mchF,	ABC transporter protein MchF	15; 50%
neuC	polysialic acid capsule biosynthetic protein	1; 3.3%
ompT	Outer membrane protease (protein protease 7)	20; 66.7%
papC,	Outer membrane usher P fimbriae	2; 6.7%
sitA,	Iron transport protein	16; 53.3%
stb	Heat-stabile enterotoxin II	3; 10%
terC,	Tellurium ion resistance protein	30; 100%
traT	Outer membrane protein complement resistance	21; 70%
tsh	Temperature-sensitive hemagglutinin	5; 16.7%
vat	Vacuolating autotransporter toxin	1; 3.3%

Supplementary Table S4: potential human pathogenicity, expressed as percentage, of ESBL *E. coli* sampled from healthy pigs and farm environment, northern Italy. Results were obtained through PathogenFinder 1.1 (https://cge.cbs.dtu.dk/services/).

Strains	Probability of human pathogenicity
**G1PAE2**	88%
**G1PAE3**	88%
**G1PAE4**	88%
**G1PAE7**	88%
**G1PAE8**	89%
**G1PAE9**	87%
**G1PHE1**	84%
**G1PHE2**	93%
**G1SAE2**	94%
**G1SAE4**	89%
**G1SAE8**	89%
**G1SAE7**	95%
**G1SAE10**	89%
**P1FAE1**	93%
**P1FAE3**	93%
**P1FAE7**	92%
**P1PAE2**	93%

**P1PAE3**	89%
**P1PAE4**	93%
**P1PAE6**	89%
**P1PAE7**	89%
**P1PAE8**	89%
**P1PAE9**	89%
**P1PAE10**	93%
**S1FHE2**	88%
**T1SAE6**	83%
**T1SAE7**	88%
**T1SAE8**	87%
**T1SAE9**	88%
**T1SAE10**	83%

Supplementary Table S5: WGS quality parameters of all analysed *E. coli* strains.

**Strains**	**Contigs**	**Reads/length reads**	**Coverage**	**N50**	**length**	**GC%**
G1PAE2	70	1006488/<br>224	46x	240995	4908608	50.45
G1PAE3	94	541164/<br>234	26x	157912	4937072	50.39

G1PAE4	63	1122266/<br>233	53x	240584	4907891	50.45
G1PAE7	151	896196/<br>220	37x	97282	5291539	50.47
G1PAE8	66	911856/<br>232	42x	209216	5057771	50.71
G1PAE9	51	1198842/<br>236	58x	208729	4852108	50.65
G1PHE1	121	787752/<br>235	40x	73509	4612127	50.61
G1PHE2	86	708166/<br>229	32x	179714	5132633	50.62
G1SAE2	161	533048/<br>235	26x	71036	4862726	50.55
G1SAE4	99	1367040/<br>224	64x	95864	4802459	50.65
G1SAE8	100	615278/<br>224	29x	91912	4801399	50.66
G1SAE10	101	1126712/<br>223	52x	95864	4802832	50.64

P1FAE1	72	1728010/<br>232	78x	209689	5110356	50.61
P1FAE3	145	1168964/<br>231	52x	111436	5175856	50.75
P1FAE7	157	2051676/<br>229	97x	76744	4837638	50.68
P1PAE2	295	432122/<br>230	20x	41072	5078038	50.52
P1PAE3	73	1084192/<br>219	45x	297184	5267640	50.42
P1PAE4	119	563930/<br>229	25x	145436	5124857	50.51
P1PAE6	97	1196434/<br>221	52x	179311	5124981	50.52
P1PAE7	167	530858/<br>226	23x	70982	5106936	50.52
P1PAE8	105	1093036/<br>218	47x	179593	5121840	50.52
P1PAE9	98	1145512/<br>225	50x	177776	5125014	50.52

P1PAE10	92	2444398/<br>219	106x	189765	5026802	50.57
S1FHE2	127	967314/<br>219	40x	176978	5316621	50.50
T1SAE6	101	845024/<br>205	36x	105200	4777837	50.77
T1SAE7	95	2079994/<br>219	94x	136287	4841785	50.68
T1SAE8	116	1029662/<br>224	51x	75014	4543562	50.81
T1SAE9	82	1074586/<br>223	50x	127839	4756721	50.60
T1SAE10	99	826014/<br>224	38x	111806	4853313	50.90

Supplementary table S6: identification of virulence and AMR genes on plasmids located on specific contigs of G1PAE2 genome. To confirm the presence of virulence and/or AMR genes on particular plasmids, tools (PlasmidFinder, ResFinder and VirulenceFinder) from Center for Genomic Epidemiology (https://cge.cbs.dtu.dk/services/) were used. NS, no significant hits.

Strain	Contig	PlasmidFinder	VirulenceFinder	ResFinder
G1PAE2	34	IncFIA 99.74 388 / 388 G1PAE2_S18_L001_R1_001_(paired,_trimmed_pairs)_contig_34 6022..6408 - AP001918	NS	NS

	31	IncFIB(AP001918) 99.56 682 / 682 G1PAE2_S18_L001_R1_001_(paired,_trimmed_pairs)_contig_31 7274..7955 - AP001918	hlyF 100 1110 / 1110 G1PAE2_S18_L001_R1_001_(paired,_trimmed_pairs)_contig_31 3116..4225 Hemolysin F UGFJ01000001<br><br>ompT 99.79 954 / 954 G1PAE2_S18_L001_R1_001_(paired,_trimmed_pairs)_contig_31 1730..2683 Outer membrane protease (protein protease 7) MG692627	NS
	36	IncFIC(FII) 95.79 499 / 499 G1PAE2_S18_L001_R1_001_(paired,_trimmed_pairs)_contig_36 24152..24648 - AP001918	cia 94.07 253 / 264 G1PAE2_S18_L001_R1_001_(paired,_trimmed_pairs)_contig_36 28783..29035 Colicin ia UGDH01000002<br><br>traT 100 831 / 831 G1PAE2_S18_L001_R1_001_(paired,_trimmed_pairs)_contig_36 11870..12700 Outer membrane protein complement resistance NC_011964	NS
	9	IncI1-I(Alpha) 99.3 142 / 142 G1PAE2_S18_L001_R1_001_(paired,_trimmed_pairs)_contig_9 9349..9490 - AP005147	NS	NS
	43	IncX1 99.2 377 / 377 G1PAE2_S18_L001_R1_001_(paired,_trimmed_pairs)_contig_43 6528..6904 - JN935898	NS	NS
	46	NS	NS	aadA1 | 100 | 792/792 | 100 | 1..792 | G1PAE2_S18_L001_R1_001_(paired,_trimmed_pairs)_contig_46 | 10820..11611 | Aminoglycoside resistance | JQ414041 | <br>aadA2b | 99.8718 | 780/780 | 100 | 1..780 | G1PAE2_S18_L001_R1_001_(paired,_trimmed_pairs)_contig_46 | 8427..9206 | Aminoglycoside resistance | D43625<br><br>sul3 | 100 | 792/792 | 100 | 1..792 | G1PAE2_S18_L001_R1_001_(paired,_trimmed_pairs)_contig_46 | 13293..14084 | Sulphonamide resistance | AJ459418

				blaCTX-M-1 | 100 | 876/876 | 100 | 1..876 | G1PAE2_S18_L001_R1_001_(paired,_trimmed_pairs)_contig_46 | 18284..19159 | Beta-lactam resistance | DQ915955<br><br>qacL | 92.2156 | 501/533 | 93.9962 | 1..501 | G1PAE2_S18_L001_R1_001_(paired,_trimmed_pairs)_contig_46 | 11681..12181 | Disinfectant resistance | NG_048048<br><br>catA1 | 99.8485 | 660/660 | 100 | 1..660 | G1PAE2_S18_L001_R1_001_(paired,_trimmed_pairs)_contig_46 | 119..778 | Phenicol resistance | V00622 | <br>cmlA1 | 99.9206 | 1260/1260 | 100 | 1..1260 | G1PAE2_S18_L001_R1_001_(paired,_trimmed_pairs)_contig_46 | 9468..10727 | Phenicol resistance | M64556
	55	NS	NS	tet(A) | 100 | 1200/1200 | 100 | 1..1200 | G1PAE2_S18_L001_R1_001_(paired,_trimmed_pairs)_contig_55 | 2349..3548 | Tetracycline resistance | AJ517790<br><br>blaTEM-1C | 100 | 861/861 | 100 | 1..861 | G1PAE2_S18_L001_R1_001_(paired,_trimmed_pairs)_contig_55 | 4041..4901 | Beta-lactam resistance Alternate name; RblaTEM-1 | FJ560503

Supplementary table S7: comparison of plasmid-positive contigs found in G1PAE2 *E. coli* strain with sequences recorded in BLAST ® database (https://blast.ncbi.nlm.nih.gov/Blast.cgi). The best match is reported in the last column on the right

Strain	Contig	Length	Best BLAST hit
G1PAE2	9	27044	plasmid G1PAE2_S18_L001_R1_001_(paired,_trimmed_pairs)_contig_9 KJ484639.1 99.945 25634 6 8 1 25626 63878 89511 0.0 47253
	31	9806	plasmid G1PAE2_S18_L001_R1_001_(paired,_trimmed_pairs)_contig_31 MG649031.1 99.959 9806 4 0 1 9806 10030 225 0.0 18087
	34	13565	plasmid G1PAE2_S18_L001_R1_001_(paired,_trimmed_pairs)_contig_34 CP056956.1 99.978 13565 3 0 1 13565 131729 118165 0.0 25034
	36	30201	plasmid G1PAE2_S18_L001_R1_001_(paired,_trimmed_pairs)_contig_36 CP056956.1 99.985 13637 2 0 16565 30201 78444 64808 0.0 25172
	43	44395	plasmid G1PAE2_S18_L001_R1_001_(paired,_trimmed_pairs)_contig_43 CP062857.1 99.955 35762 16 0 8634 44395 44395 8634 0.0 65952
	46	22534	plasmid G1PAE2_S18_L001_R1_001_(paired,_trimmed_pairs)_contig_46 CP026933.2 100.000 15917 0 0 20 15936 176072 160156 0.0 29394
	55	5785	plasmid G1PAE2_S18_L001_R1_001_(paired,_trimmed_pairs)_contig_55 OW848878.1 100.000 4445 0 0 1341 5785 13217 8773 0.0 8209
